# Solvent Effect on Antimicrobial Hydrophilic Xerogel Coating of Medicinal Leathers in Simulated Industrial Finishing Process

**DOI:** 10.1002/cplu.202400648

**Published:** 2025-02-21

**Authors:** Theofanis Bompotis, Eirini Karastergiou, Konstantinos Giannakopoulos, Evangelos P. Favvas, Marina Arvanitopoulou, Konstantinos Arvanitopoulos, Labros Arvanitopoulos, Georgia Kytherioti, Michail Vardavoulias, Dimitrios A. Giannakoudakis, Laura Castellsagués, Sara Maria Soto González., Michael Arkas

**Affiliations:** ^1^ Institute of Nanoscience Nanotechnology, NCSR “Demokritos” Patriarchou Gregoriou Street 15310 Athens Greece; ^2^ DARVICHEM Alexandrou Papagou 5 18233 Agios Ioannis Rentis Greece; ^3^ Institute of Bioscience and Applications NCSR “Demokritos” Patriarchou Gregoriou Street 15310 Athens Greece; ^4^ Department of Science and Mathematics School of Liberal Arts and Sciences The American College of Greece, Deree Gravias 6 15342 Athens Greece; ^5^ PYROGENESIS S.A., Technological Park 1 Athinon Avenue 19500 Attica Greece; ^6^ Faculty of Chemistry Maria Curie-Skłodowska University Maria Curie-Skłodowska Sq. 3 20-031 Lublin Poland; ^7^ Barcelona Institute for Global Health (ISGlobal) Universitat de Barcelona 08036 Barcelona Spain; ^8^ CIBER Enfermedades Infecciosas (CIBERINFEC) Instituto de Salud Carlos III 28029 Madrid Spain

**Keywords:** dendrimers, leathers, sustainable chemistry, antibacterial, hydrophilicity

## Abstract

The hydrophilic character and the protection against pathogen proliferation are the most pivotal characteristics of leathers intended for medical purposes. To achieve these goals, dispersions of TiO_2_ particles incorporating three different formulations of biomimetically synthesized silica xerogels were tested. Emphasis has been given to the role of single and dual solvents employed. Microbiocide capability was induced by benzalkonium chloride along with silver nanoparticles. Particular emphasis should be given to hyperbranched poly(ethylene imine) multifunctional roles. Spontaneous mineralization of silver ions is realized in the dendritic cavities. The same polymer acts as a matrix that interacts with the hydrogen bonding network of orthosilicic acid directing and facilitating gel formation. Furthermore, it contributes to both hydrophilicity and antimicrobial properties. Gel formation and subsequent drying occur in the pores of the impregnated TiO_2_ substrate. The resistance of the leathers to fungal and bacterial infections and biofilm formation was assessed against *Klebsiella Pneumoniae, Escherichia coli*, *Pseudomonas aeruginosa, Enterococcus faecalis, Staphylococcus aureus*, and *Candida albicans*. The affinity to water was proved by the contact angle method. The proposed treatment is a prospective environmentally friendly replacement to the standard finishing process of medical leathers.

## Graphical abstract

Silica‐hyperbranched poly(ethylene imine) (PEI) xerogels incorporating silver nanoparticles coated titanium dioxide particles. Dual solvent dispersions of these composites enhance medical leathers′ hydrophilicity and antimicrobial properties. The nucleation of the metal ions into the dendritic polymer‘s cavities and the gel formation were inspired by biological processes and are performed at ambient temperature, aqueous solvents, without toxic byproducts.

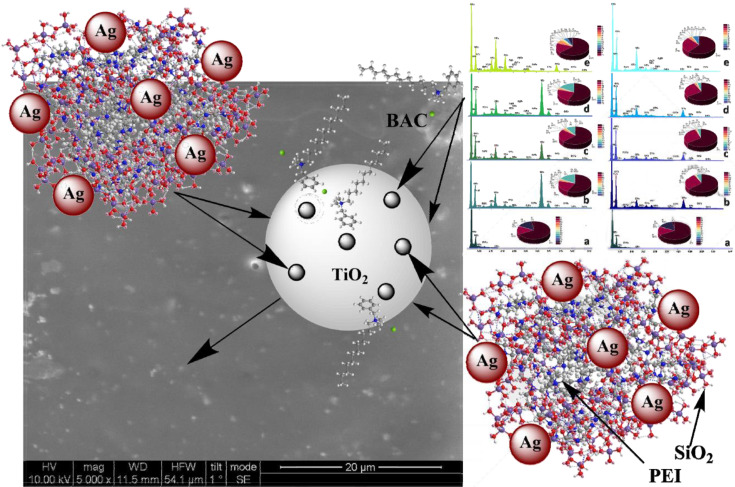

## Introduction

The treatment of hides, one of the oldest technologies developed by hominids, at least Neanderthals and Humans, dates from the Paleolithic era. Evidence of hide working has been found from about 400,000 years ago.^[1]^ The first tanning reagent was believed to be red ochre.^[2]^ Protection against low temperatures, animal attacks, and other adverse environmental challenges was a basic necessity and a crucial catalyst for humanity‘s expansion throughout Earth.

Over the centuries the implementations of leather spread from footwear, clothes handbags, other clothing accessories, and furniture to bookbinding automotive interiors and sporting items. The development of medical counterparts proceeded in parallel. Wheelchairs, alternating pressure mattresses, intensive care and hospital furniture, protective gear, and footwear require antimicrobial and antibiofilm protection. Moreover, specialized products need increased hydrophilicity to become water vapor permeable and/or to host polar active ingredients. Some examples include antiallergic and abrasion resistance agents, dyes, and volatile compounds that induce the characteristic leather scent.^[3]^ To fulfill all these perquisites and obtain a final product of the highest quality the raw hides need to undergo a series of processes that involve a variety of environmentally harmful compounds such as heavy metals predominantly chromium at its highest oxidation state, polychlorinated long‐ chain saturated hydrocarbons and perfluorinated analogs.[^4^, ^5^]

Moreover, leather cleaning is a further source of trouble that needs to be resolved. Generally, conventional washing machines and detergents are not recommended. A promising alternative followed by modern finishing techniques is the decomposition of the organic impurities. To this end, photolytic processes are considered. Titania (TiO_2_) is applied not only due to its well‐known photocatalytic^[6]^ and conventional catalysis properties^[7]^ but also because it exhibits hydrophilicity^[8]^ and thus inhibits the reaccumulation of lipophilic litter. Moreover, it is an effective antibacterial agent^[9]^ and therefore capable of self‐ cleaning and sterilizing purposes.[^10^, ^11^] For these reasons, TiO_2_ is a finishing agent during the hides treatment process.^[12]^

Both the photocatalytic^[13]^ and antibacterial potential^[14]^ of TiO_2_ are enhanced in the presence of silver nanoparticles (Ag NPs) since they represent an ideal substrate for their homogenous distribution.^[15]^ The Ag NPs are very potent bactericides for a broad spectrum of microorganisms due to their high surface concerning their overall volume.[^16^, ^17^, ^18^] In an emulsified or colloidal form[^19^, ^20^] or encapsulated into suitable formulations^[21]^ they may replace chromium‘s role in the inhibition of microbe proliferation to leather and fur substrates as well as serve in medical implementations as wound dressings^[22]^ and catheters.^[23]^

The best strategy to develop uniformly dispersed Ag NPs ecologically is to get inspired by natural processes and more specifically by the action of biomineralization proteins such as ferritin in microorganisms like *Pyrococcus furiosus archaea*.[^24^, ^25^] To mimic their operation, dendritic polymers represent a category of prominent compounds. The radial polymerization used for their construction^[26]^ apart from the differentiation from the other polymeric types induces an architecture similar to a tree's branches.[^27^, ^28^, ^29^, ^30^, ^31^] These patterns, particularly at the molecule‘s interior, create cavities susceptible to adaptations to absorb and host various compounds.[^32^, ^33^] In this way, higher concentrations of active compounds and dendritic branching/functional groups are achieved in limited space. Consequently, the polymeric matrices act as nanoreactors that do not follow the typical chemical rules and restrictions that apply to conventional solutions. Symmetrical dendrimers like poly(amido amine) (PAMAM) and hyperbranched polymers like poly(ethylene imine) (PEI) bearing nitrogen functionalities resemble the size and properties of proteins. They are ideal alternatives for nucleating metal,^[34]^ specifically silver[^35^, ^36^] nanoparticles. Moreover, PEI is non‐symmetrical, thus cheaper, and contributes to the coating formulations′ hydrophilic and antimicrobial properties.^[37]^

For the incorporation of the Ag NPs into the pores of the TiO_2_ substrate we are taking advantage of the terminal amino groups at the periphery of PEI to intervene in the hydrogen bond network of orthosilicic acid and form composite hydrogels. This mediation facilitates the conversion of the silanol groups to siloxane bridges of silica replicating the action of another type of protein the silaffins. Water removal by mild heating or vacuum affords xerogels.[^38^, ^39^, ^40^] Silica‐PEI xerogels are excellent adsorbents and implementations in lanthanide^[41]^ and actinide[^42^, ^43^] removal‐recovery are already being researched. The fusion of mineralization and silicification^[44]^ yields multifunctional hybrid ceramic materials incorporating the dendritic polymer matrices bearing metal nanoparticles that have already been applied in many medical fields.[^45^, ^46^] The gel precursor solution apart from the surface may also be readily adsorbed through the pores of the TiO_2_ particles and diffuse homogenously to its entire volume. After the solidification/gelation and the subsequent drying; composite TiO_2_ particles bearing silica‐PEI‐Ag NPs xerogels are produced. In the form of dispersion, they proved capable of meeting the specifications of conventional titania powder and hosting an optional supplementary active ingredient. For this purpose, we delved into a prominent class of biocides equally notable for the diversity of their self‐organization properties salts bearing quaternary nitrogen groups.^[47]^ Among these, a commercial mix of benzyl dimethyl ammonium chlorides with a fourth‐long aliphatic chain of variable length typically used in textiles: Benzalkonium chloride (BAC) represents the most obvious candidate.^[48]^ Silver nanoparticles in the solid state as inert inorganic materials are less susceptible to degradation than organic BAC. Both active ingredients though are expected to remain active for the entire period of the substrate's use.

As a first proof of concept, Ag NPs and their dendritic PEI matrix were combined with orthosilicic acid. The gel precursor solution impregnated bovine leathers and gelification took place in the interior of the porous substrate increasing its polarity and thus absorption of BAC. The resulting coated samples effectively inhibited bacteria proliferation and biofilm formation whereas cell cultures remained unaffected. The latter was attributed to silanol counterions eliminating the high local concentrations of toxic cationic ammonium species.^[49]^ As a first adaptation of the concept to a commercially feasible formulation the above‐ described gel formation was performed in the pores and the surface of TiO_2_ microparticles with a posterior addition of BAC at a final stage. A single solvent dispersion was generated to coat leather samples from various animals and equally promising results were obtained.^[50]^ In the current third implementation, we investigate the applicability of the xerogel dispersions in the standard leather finishing process parameters focusing mainly on the effect of dual‐solvent dispersions.

## Results and Discussion

### Stability of Coatings and Leather Components

Ammonium salts are generally thermally stable in the solid state below 150 °C either primary^[51]^ or quaternary.^[52]^ The other components of the dispersion powders can withstand far higher temperatures. However, in dispersion in aqueous solutions and temperatures above 45 °C, a small quantity of organic content is released most probably ethanol remains from the hydrolysis of tetraethoxysilane.^[43]^

**Figure 1:**
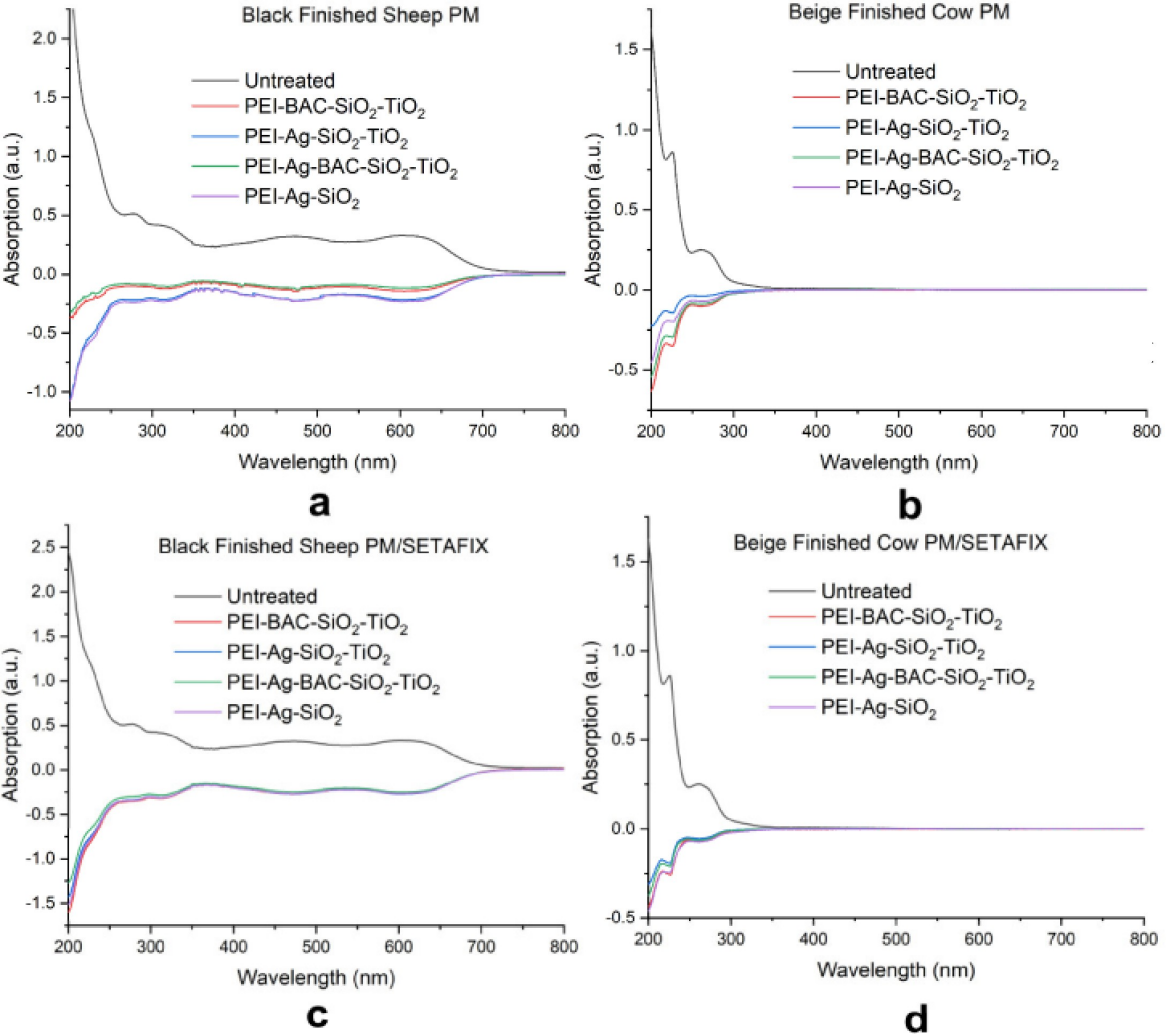
Comparison of UV‐Vis spectra of supernatants of untreated leathers after 1‐week immersion into the water with differential spectra of the coated samples obtained using corresponding supernatants of untreated leathers as controls. (a) Black Finished Sheep treated by PM dispersion (b) Beige Finished Cow treated by PM dispersion (c) Black Finished Sheep treated by PM/SETAFIX dispersion (d) Beige Finished Cow treated by PM/SETAFIX dispersion.

The first evaluation of the prepared samples comprised the assessment of the diffusion of the components of the coatings into the water and additionally involved a direct comparison of their overall stability with the uncoated precursors. Both treated and untreated specimens (0.5×0.5 cm) were placed into 10 ml of ultrapure water and allowed for one week in an orbital shaker. Then the standard UV‐Vis spectra of the supernatants of the original leathers (i. e. having water at the control compartment) (Figure 1 Black lines) were superimposed over differential spectra of the respective solutions of the treated samples obtained with the supernatant of the corresponding uncoated sample at the control compartment (Figure 1 colored lines). The spectra of both cow and sheep leathers are characterized by wide and intense absorption bands at the zone of UV light (200–350 nm) indicating a substantial diffusion of their organic content to water. The Black Finished Sheep exhibits additional absorption peaks in the visible region suggesting decolorization.

All the derivative spectra measured for the coated samples are directed downwards. This is caused by the smaller release of the leather‘s components. Therefore, leather processing with the dispersions of composite titania particles containing the hybrid xerogels delays decay and decolorization, or at least those caused by contact with humidity and water. A more elevated protection is observed for Black finished Sheep samples and the dual solvent system PM/SETAFIX (Figure 1c) regardless of the composition of the dispersed powder. This combination additionally effectively inhibits decolorization. Since there are no peaks in the positive section of these spectra diffusion of absorbing substances such as Ag NPs (410 nm) present in the dispersions and not in the original leathers must be excluded. Moreover, the baselines of all samples either treated or untreated are identical. The absence of an upward shift means that there is no turbidity connoting that there is no redispersion of the titania or the xerogel powders to water.

**Figure 2:**
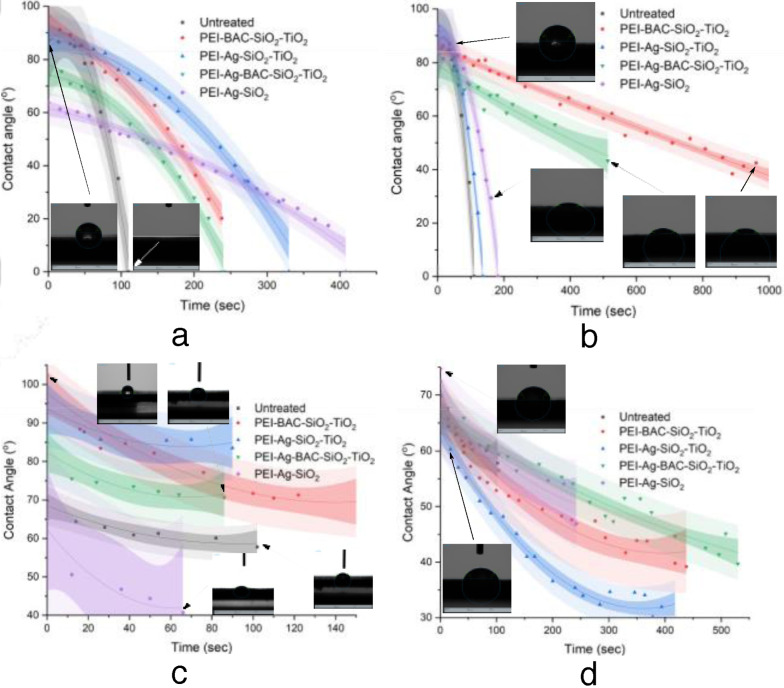
The hydrophilicity of the leather sample surfaces is expressed as a decrease in the contact angle of a water drop as a function of time. (a) Beige Finished Cow treated solely by PM; (b) Beige Finished Cow treated by SETAFIX; (c) Black Finished Sheep treated solely by PM; (d) Black Finished Sheep treated by SETAFIX; Dark and light shadowy areas represent 95 % Confidence and Prediction bands respectively.

A substantial number of both treated and untreated substrates adsorbed several drops of water without attaining equilibrium. For this reason the variation of contact angle as a function of time is reported. As can be seen in Figures 2a and 2b uncoated cow samples present an excellent capacity to absorb completely the first water drop after about 100 seconds. Applying a simple xerogel coating (i. e. without the titania powder) does not increase WCA yet adsorption is delayed most probably due to pore clogging. Generally, treatment with PM increases absorption/penetration time by up to 4 times. On the other hand, the employment of SETAFIX has only a slight delay effect on the samples that do not contain BAC. It seems though that it is not compatible with BAC taking into account the dramatic increase in the water drop absorption/penetration time. Most probably this BAC‐SETAFIX interaction in conjunction with the presence of the TiO_2_ induces some pore clogging.

To assess the durability of the dispersion powders to the UV irradiation two samples of PEI‐Ag‐SiO_2_‐TiO_2_ were added in aqueous solution (10 mg in 10 ml) and sonicated for one hour to form a milky dispersion. The one was irradiated at 252 nm for one hour. Subsequent macroscopic evaluation did not show any difference (Figure S1). A more detailed investigation by UV‐visible spectroscopy revealed a slight negative deviation in the turbidity of the irradiated sample. Both specimens did not exhibit substantial absorption between 200–300 nm indicating no diffusion of the organic content of the composites to water. A measurement of the non‐irradiated dispersion with the irradiated analog as the reference sample resulted in the same difference in turbidity and a small peak near 200 nm attributed most probably to the higher turbidity of the non‐irradiated sample.

### Hydrophilicity of the Leather Samples

The affinity of the leather surfaces to water is a critical factor affecting the capability of the final products to incorporate all kinds of polar substances. Typically, it is expressed by equilibrium water contact angle (WCA). The increase in hydrophilicity by coating leathers with xerogels^[49]^ and titania‐ silica xerogel dispersions^[50]^ is already established in hydrophobic bovine leathers with water contact angles in the order of 100–113°. The main scope of the current series of experiments is to ensure that hydrophilicity is not compromised by the introduction of the PM‐SETAFIX as a dispersion solvent.

The respective sheep specimens are a bit more hydrophobic establishing an equilibrium at a water contact angle of about 60°. In this case, too PM renders surfaces coated by titanium dispersions more hydrophobic (Figure 2c). In contrast, the addition of SETAFIX has the same beneficial effect on the hydrophilic character of the surface as above. This is more profound in the samples that do not contain BAC (Figure 2d).

### SEM and EDS Analysis

**Figure 3:**
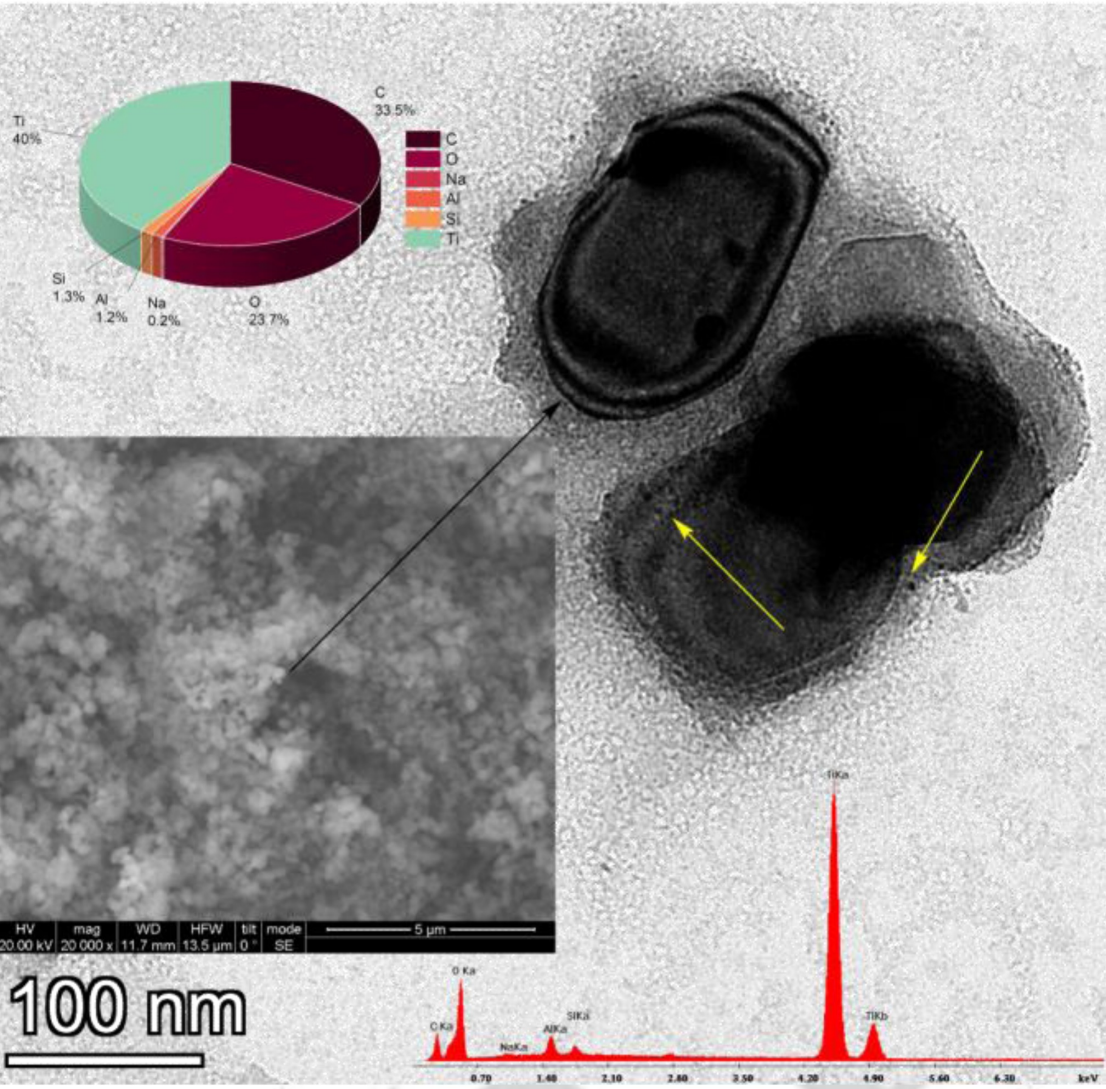
SEM (insert) and TEM images of the PEI‐Ag‐SiO_2_‐TiO_2_ composite. In the TEM picture dark areas at the core of the particles represent TiO_2_ lighter, grey areas are SiO_2_ xerogel and the small black spots indicated by the yellow arrows are silver nanoparticles.

Titania is comprised of quasi‐spherical particles as shown in the SEM micrograph (inset of Figure 3). A more detailed depiction by TEM microscopy reveals that the dark ΤιΟ_2_ cores exhibit a certain crystallinity and are surrounded by a light grey entourage corresponding to the silica xerogel. This means that xerogel is not absorbed only into the pores by also coats homogenously and almost completely the TiO_2_ substrate. At the interphase between SiO_2_ and TiO_2_, there are some sparse black spots most probably attributed to silver nanoparticles. The overall silver content of the samples is too low to permit the calculation of a size distribution (below 0,1 %) and the characteristic EDS peaks of Ag are not distinguished from the background. The Silver nanoparticles are smaller than 5 nm as calculated in solution by DLS (i. e. along with their solvation spheres).^[35]^ A rough approximation of their size is about 3 nm. The weight ratio SiO_2_/TiO_2_ is 1/29 lower than the theoretically predicted (1/25) corroborating the hypothesis that part of the silica xerogel has entered the pores.

**Figure 4:**
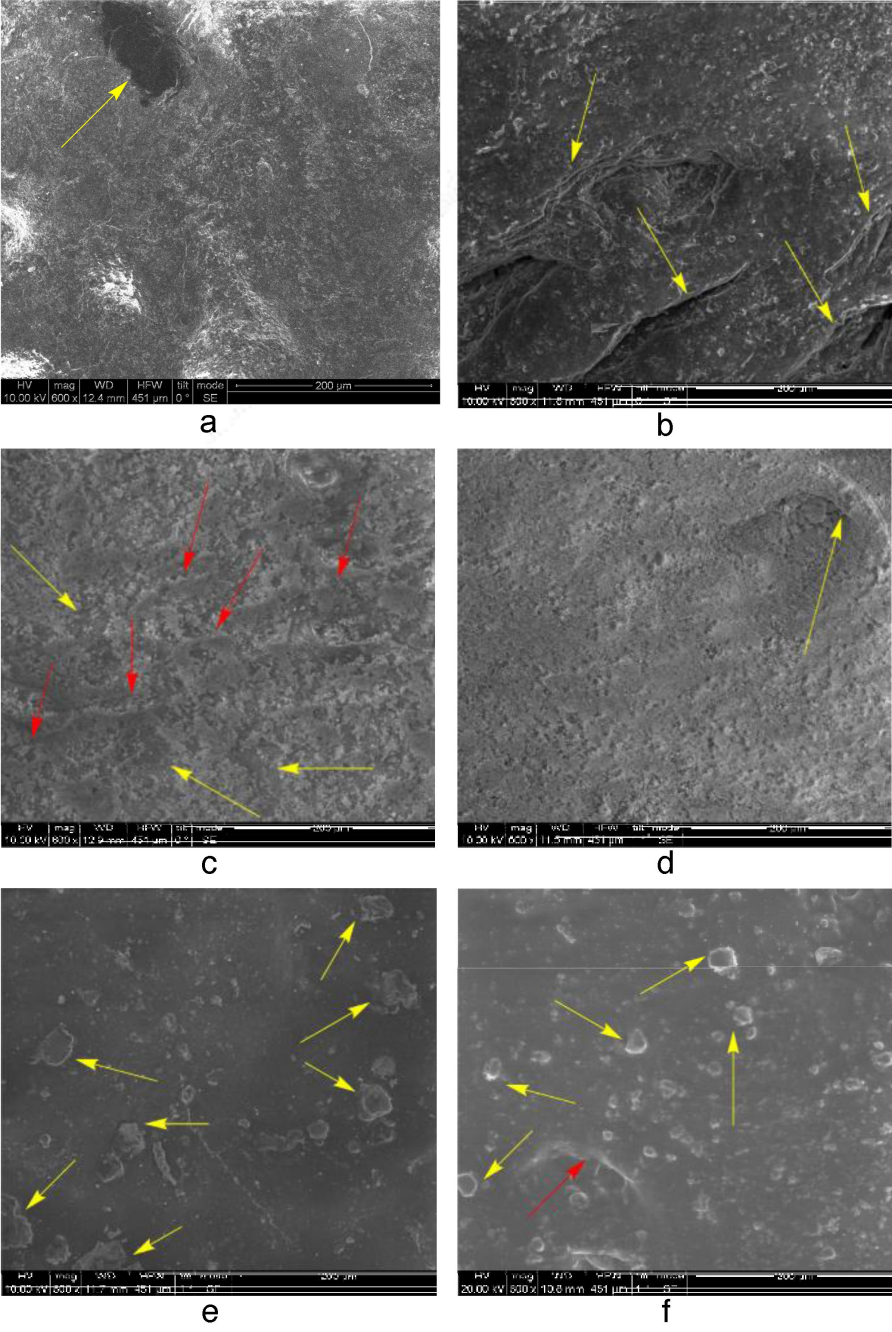
SEM micrographs of untreated Black Finished Sheep leather (a) and Beige Finished Cow (b) treated with PEI‐BAC‐SiO_2_‐TiO_2_ in PM (c and d) respectively), and PM/SETAFIX dispersions (e and f respectively).

All SEM micrographs of the coated specimens were obtained from 1 cm^2^ square coupons under low vacuum conditions since high vacuum application deformed the samples. Pores (Figure 4a) and other irregularities, such as crumples (Figure 4b), are recognizable on the surface of untreated sheep (Figure 4a) and cow substrates (Figure 4b). Their overall coarseness and general heterogeneity are also evident.

Coating by the same composition, for instance, PEI‐BAC‐TiO2‐SiO2 in single PM dispersions covers the cow leather samples more homogeneously compared to the sheep counterparts. In the latter uncoated parts appear as darker areas (Figure 4c, yellow arrows) among the slightly visible folds, (Figure 4c, orange arrows). Please check the corrected figure 4a in the pdf These darker sections are absent from the respective cow samples. In contrast, the coating powder‘s partial infiltration of the pores is also observed (Figure 4d) corroborating the hypothesis that the decrease of water permeation observed for the BAC‐containing samples is attributed to pore clogging.

The more efficient layering is probably attributed to the higher hydrophilicity of the underlying substrate, which allowed for a better diffusion of the polar dispersion. Treatment with PM/SETAFIX dispersions enhances the layers’ width (Figures 4e, 4f). The underlying features (pores and folds) previously partially concealed under PM are now completely obscured or scarcely visible, like the pore indicated by the orange arrow in Figure 4f. It also causes the formation of agglomerations (Figures 4e, 4f yellow arrows).

**Figure 5:**
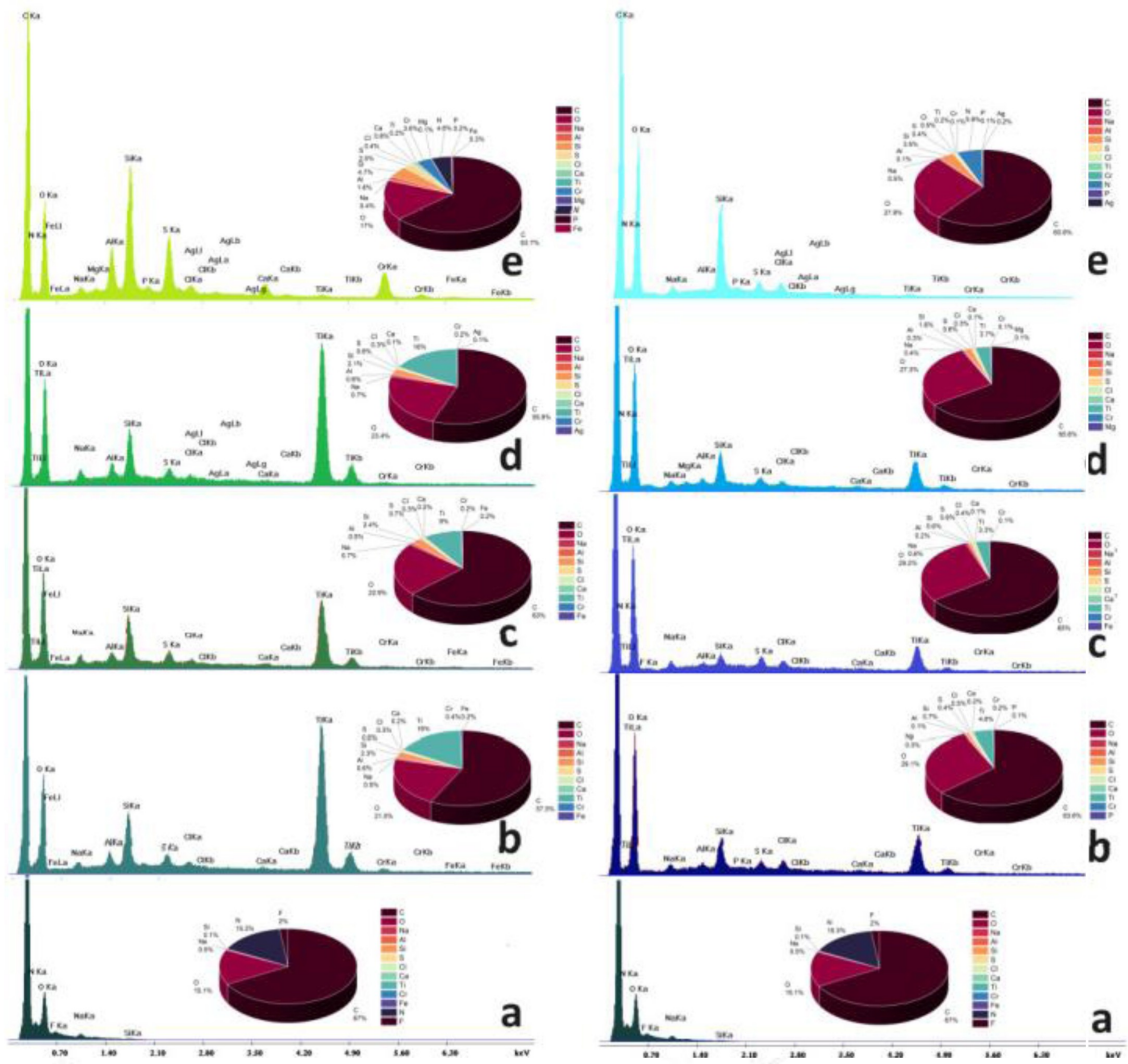
EDS spectra of Sheep leather (a) untreated and treated with PM (left) and PM/SETAFIX dispersions (right) (b) PEI‐BAC‐SiO_2_‐TiO_2_ (c) PEI‐Ag‐SiO_2_‐TiO_2_, (d) PEI‐Ag‐BAC‐SiO_2_‐TiO_2_ and (e) PEI‐Ag‐SiO_2_.

**Figure 6:**
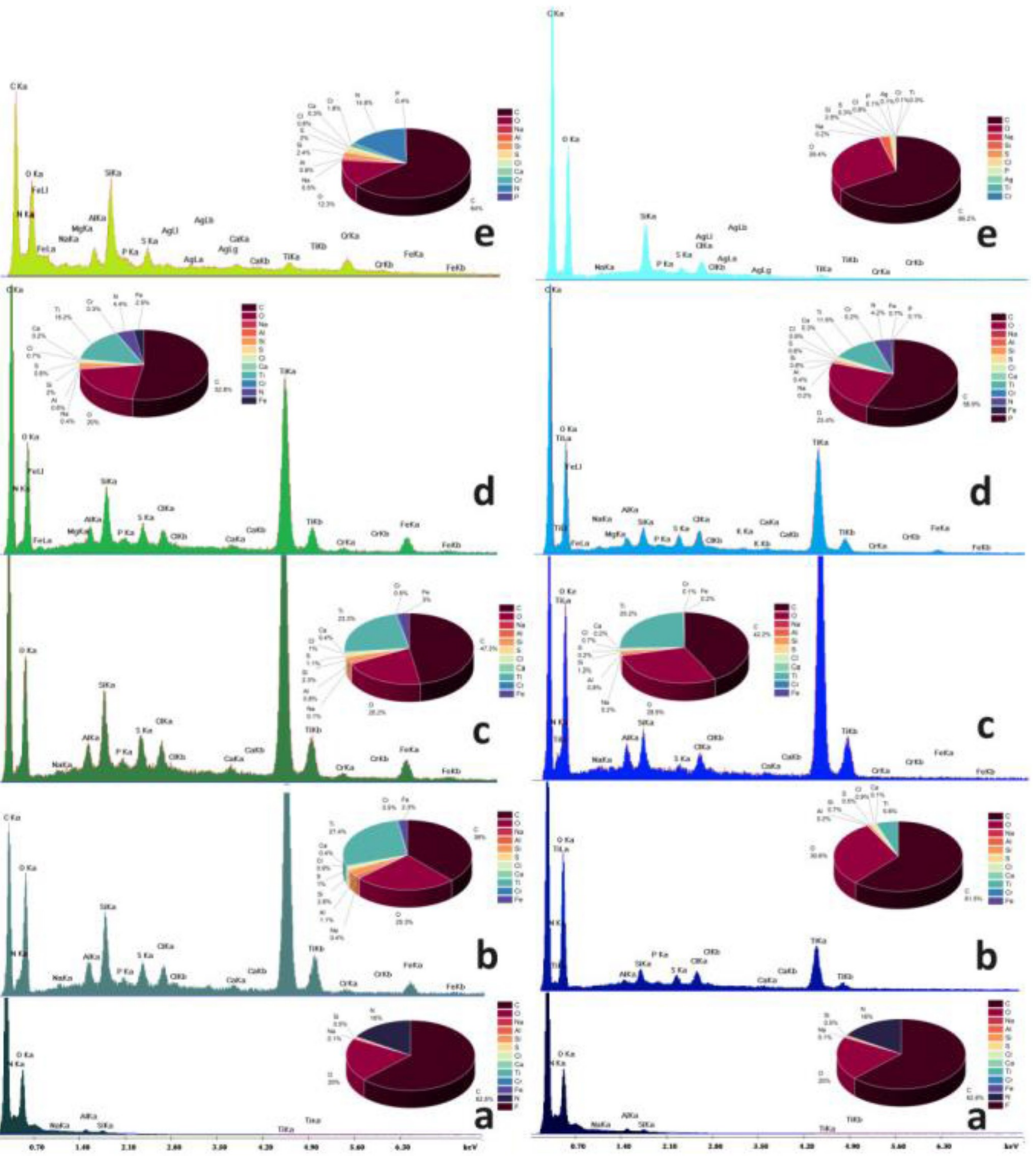
EDS spectra of Cow leather (a) untreated and treated with PM (left) and PM/SETAFIX dispersions (right) (b) PEI‐BAC‐SiO_2_‐TiO_2_ (c) PEI‐Ag‐SiO_2_‐TiO_2_, (d) PEI‐Ag‐BAC‐SiO_2_‐TiO_2_ and (e) PEI‐Ag‐SiO_2_.

The energy‐dispersive X‐ray (EDS) spectra of Sheep leather samples either untreated or coated with silica or silica/titania dispersions are shown in Figure 5. The respective Cow analogs are displayed in Figure 6. Both raw specimens have about the same carbon (63–67 %), oxygen (15–20 %), and nitrogen (15–16 %) percentages corresponding to keratin collagen and the other common hide proteins. The sodium traces represent the remains of NaCl employed for dehydration as a preservative of the leather proteins and as a bacteriostatic. Moreover, the denaturation of interfibrillar proteins is performed by Na_2_S. Small quantities of fluorine originate from the perfluorinated derivatives used to increase resistance to water. Silicon and magnesium are present due to sodium aluminosilicate (AlNa_12_SiO_5_) and MgO used for re‐tanning and kaolinite (Al_2_O_3_ 2SiO_2_⋅2H_2_O) employed during bating. Traces of elements belonging to reagents employed in the other stages of transformation of raw hides to commercial products are also found mainly in the EDS spectra of the coated samples. Besides aluminum which was justified above sulfur (in addition to Na_2_S) and chromium are elements composing the reagent employed for the tanning process: ([Cr(H_2_O)_6_]_2_(SO_4_)_3_). Iron salts with sulfur and chlorine represent an alternative tanning reagent. The collagen fibers are stabilized during liming by Ca(OH)_2_.

Processing with titania dispersions causes the appearance of the TiKa (4,5 keV) and (TiKb 5 keV) characteristic peaks corresponding to percentages 9–27 % at the samples that underwent the PM process and 3–25 % for the PM/SETAFIX dispersions. This is normal since the PM/SETAFIX method assumes more dilute dispersions (see experimental section). Cow surfaces exhibit greater affinity to titania retaining larger Ti percentages (5–27 %) in comparison to sheep (3–16 %).

Silicon content is also upgraded and this is reflected in the enlargement of the SiKa peak at 1,73 keV establishing the presence of silica xerogels at the surface and into the pores of the titanium oxide. In some cases, evidence of the presence of silver nanoparticles can be found as AgLI (2.8 keV), AgLa (3 keV) AgLb (3.1 keV), and AlLg (3.5 keV) peaks.

As expected the samples coated by simple xerogels are characterized by stronger SiKa peaks. This same effect applies to the Ag Nps peaks.

### Antimicrobial and Antibiofilm Studies

#### Inhibition‐Zone Method

**Figure 7:**
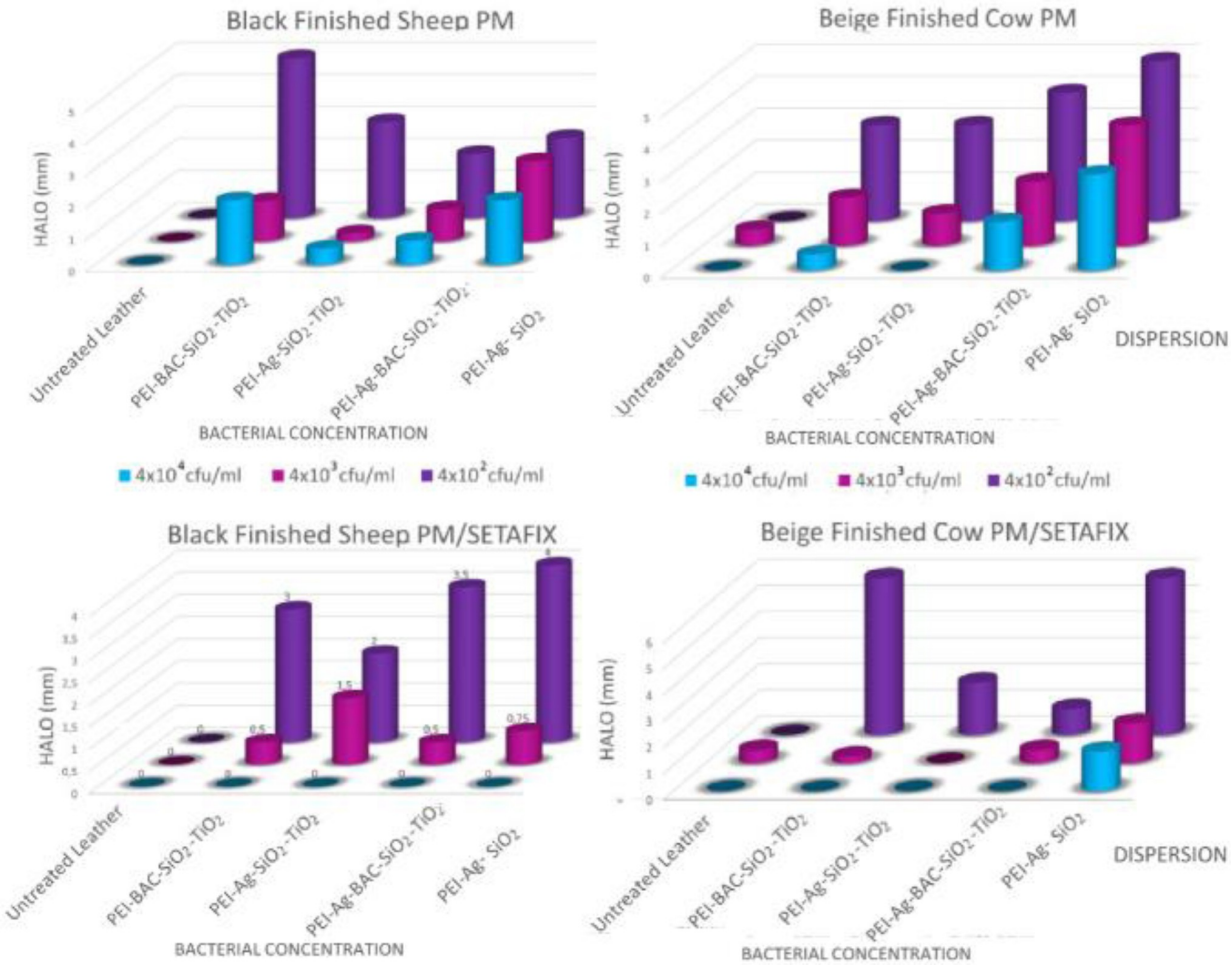
Zones of inhibition of *Escherichia coli* proliferation around leather samples untreated and treated with the 4 xerogel powders at 3 different bacteria concentrations (a) Black Finished Sheep PM dispersion (b) Beige Finished Cow PM dispersion (c) Beige Finished Cow PM/SETAFIX dispersion (d) Black Finished Sheep PM/SETAFIX dispersion.

**Figure 8:**
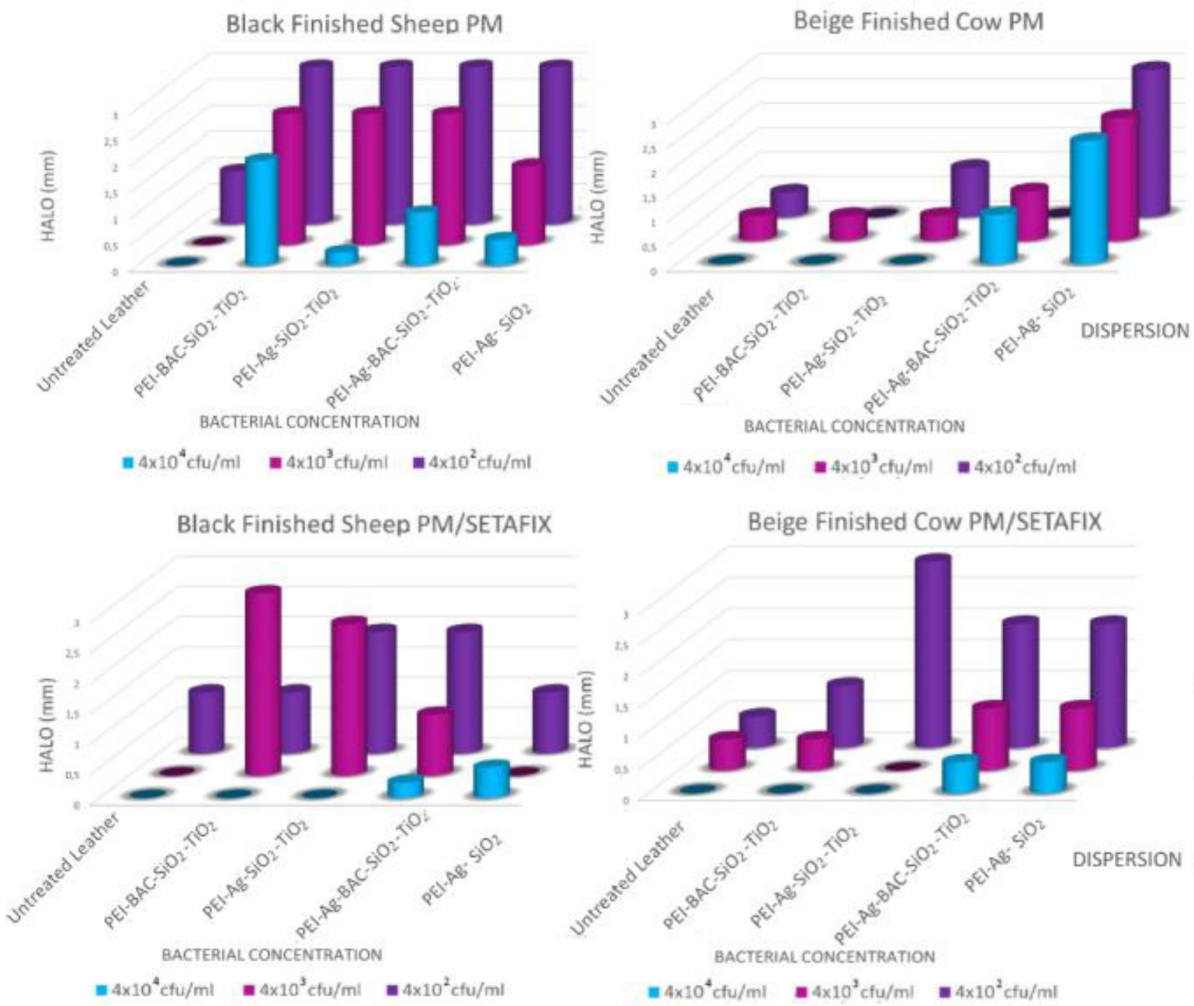
Zones of inhibition of *Pseudomonas aeruginosa* proliferation around leather samples untreated and treated with the 4 xerogel powders at 3 different bacteria concentrations (a) Black Finished Sheep PM dispersion (b) Beige Finished Cow PM dispersion (c) Beige Finished Cow PM/SETAFIX dispersion (d) Black Finished Sheep PM/SETAFIX dispersion.

**Figure 9:**
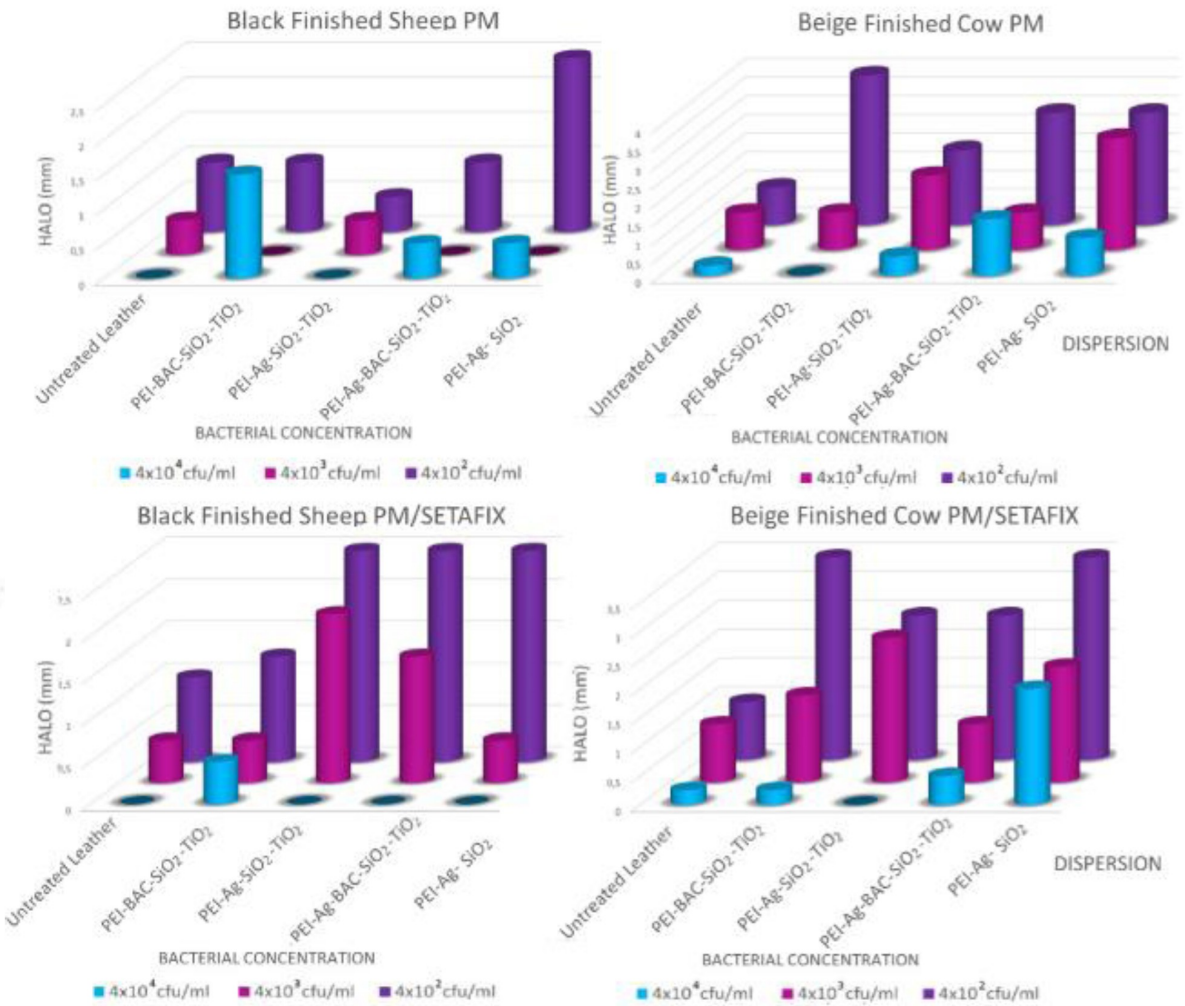
Zones of inhibition of *Staphylococcus aureus* proliferation around leather samples untreated and treated with the 4 xerogel powders at 3 different bacteria concentrations (a) Black Finished Sheep PM dispersion (b) Beige Finished Cow PM dispersion (c) Beige Finished Cow PM/SETAFIX dispersion (d) Black Finished Sheep PM/SETAFIX dispersion.

To obtain an initial approximation of the antimicrobial protection of the coatings cultures of three different concentrations *of Escherichia Coli*, *Staphylococcus aureus*, and *Pseudomonas aeruginosa* were layered in Luria–Bertani (LB) medium plates. A comparison of the areas around the coated samples where bacterial colonies could not be observed with those produced by the untreated counterparts gave a good estimation of their antimicrobial activity. As can be deduced from the diagrams of Figures 7, 8, and 9 untreated samples present minimal halos up to 1 mm and only in the cases of the smaller bacterial concentrations. Both are more resistant to *Staphylococcus aureus* and *Pseudomonas aeruginosa*. In contrast inhibition zones up to 6 mm appear on the plates of the treated samples with optimum performance observed against *Escherichia Coli*. As normally anticipated the biggest bacterial concentrations result in the smallest inhibition zones. Except for one case of Beize Finished Cow samples treated only by PM dispersions and infected with *Pseudomonas aeruginosa*, the protection offered by the PEI‐Ag‐SiO_2_ xerogels is comparable to that of the xerogels formulations that were formed into the pores of the TiO_2_ powder. This means that the concept of the gelation and drying of the xerogels into the pores of titania is feasible. Interestingly BAC seems more effective against *Escherichia Coli* and *Staphylococcus aureus* while Ag NPs inhibit drastically *Pseudomonas aeruginosa*. The formulation that contains both active ingredients presents a good overall solution for all the microorganisms. On the other hand, if BAC's toxicity needs to be avoided Ag NPs may reliably provide an adequate alternative.

**Figure 10:**
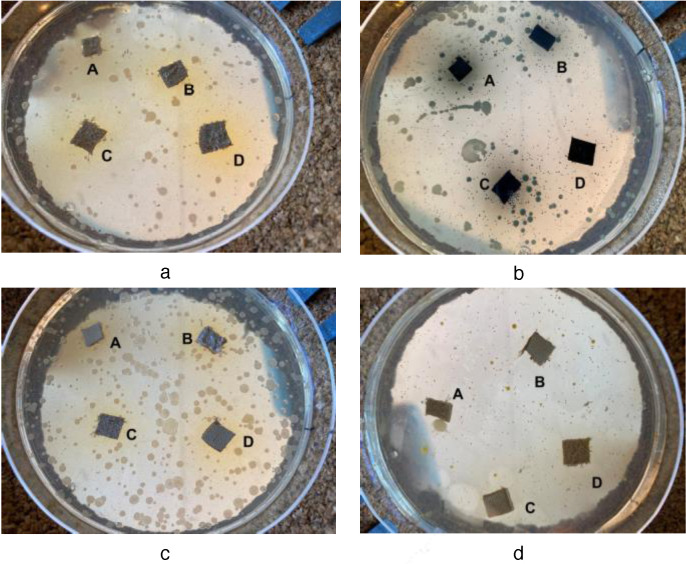
The proliferation of 4x10^4^ cfu/ml bacteria onto selected LB‐medium agar plates. The four leather coupons immersed into each plate are treated by A: PEI‐BAC‐SiO_2_‐TiO_2_, B: PEI‐Ag‐SiO_2_‐TiO_2_, C: PEI‐Ag‐BAC‐SiO_2_‐TiO_2_, D: PEI‐Ag‐SiO_2_ dispersions (a) *Pseudomonas aeruginosa*, Beige Finished Cow, PM Dispersion (b); *Staphylococcus aureus*, Black Finished Sheep, PM Dispersion (c) *Pseudomonas aeruginosa*, Beige Finished Cow, PM/SETAFIX Dispersion (d); *Staphylococcus aureus*, Black Finished Sheep, PM/SETAFIX Dispersion.

In the lower bacterial concentration (4×10^2^ cfu/ml) the dual solvent dispersions equal and in many instances outperform the results of the single solvent counterparts. In the higher concentrations of 4×10^3^ cfu/ml and particularly 4×10^4^ cfu/ml their effectiveness presents a hysteresis for the Gram‐negative *Pseudomonas aeruginosa* and *Escherichia Coli* (Figures 10a, 10b) whereas the results for the Gram‐positive *Staphylococcus aureus* are a bit better **(**Figures 10c, 10d). Nevertheless, the coatings for both dispersion categories protected the leather coupons against all types of bacteria. The dual solvent dispersion option may be effectively employed with the TiO_2_ powders bearing the composite xerogels in the hide treatment process. Finally, it should be noted that the inhibition zones were generally limited compared to those around leather samples directly coated by the xerogel precursor solution.^[49]^ Given that the quantities of the latter coatings were about tenfold higher; promising perspectives arise. For instance, using denser dispersions or TiO_2_ powder with higher content in xerogels is expected to give superior effects.

### Protection Against Adhesion of Microbial Colonies

**Figure 11:**
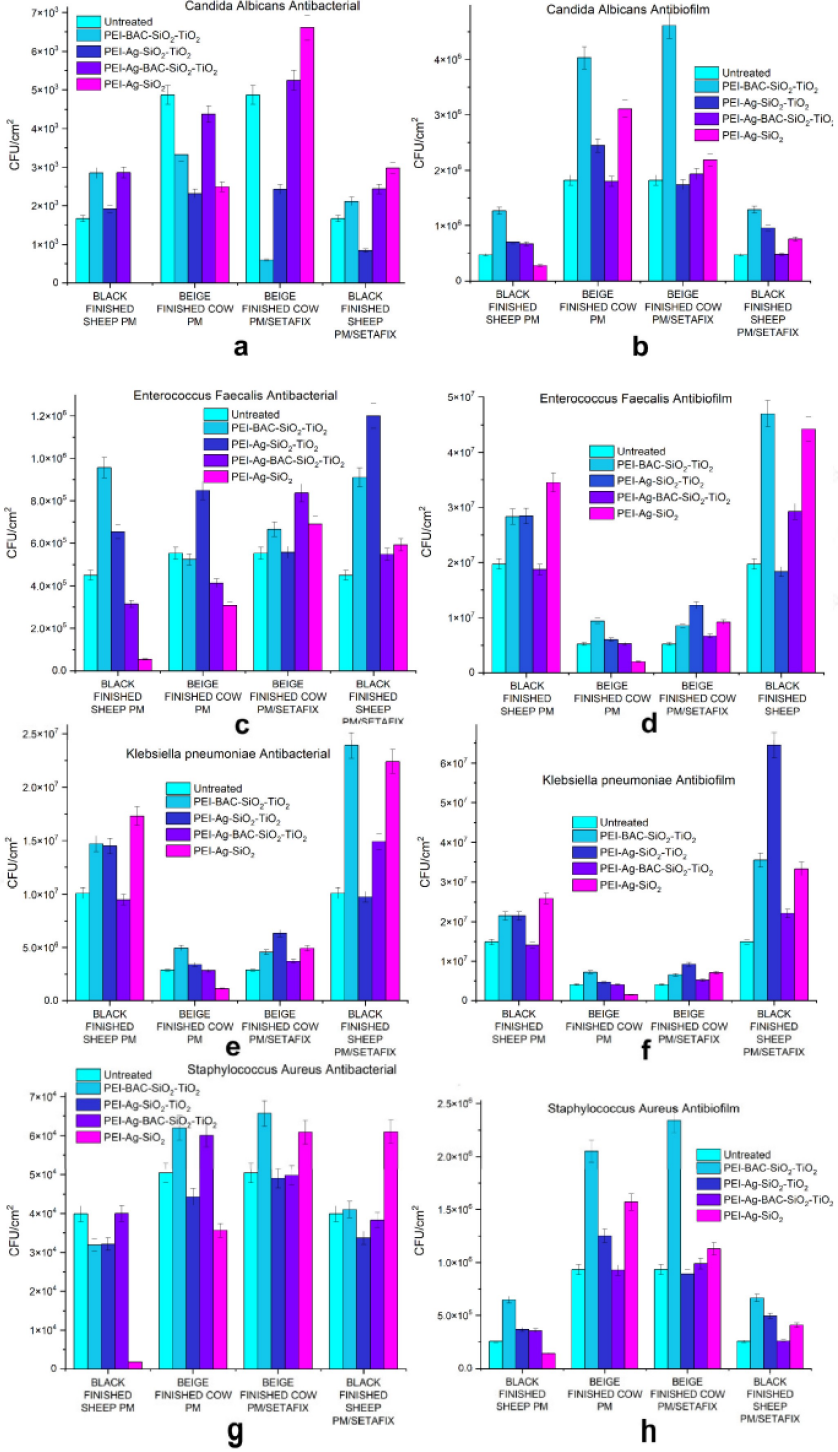
Microbial (bacterial‐fungal) colonies developed on the xerogel‐ treated leather substrates (a) *Candida albicans* after 2 hours of incubation. (b) *Candida albicans* after 48 hours of incubation. (c) *Enterococcus faecalis* after 2 hours of incubation. (d) *Enterococcus faecalis* after 48 hours of incubation. (e) *Klebsiella pneumoniae* after 2 hours of incubation. (f) *Klebsiella pneumoniae* after 48 hours of incubation and (g) *Staphylococcus aureus* after 2 hours of incubation. (h) *Staphylococcus aureus* after 48 hours of incubation

In the next test round, the adhesive forces between a higher concentration (5×10^5^ cfu) of microbial colonies and treated leather surfaces were investigated (Figure 11). Simple composite xerogel PM dispersions effectively inhibited the formation of colonies of all microorganisms except *Klebsiella pneumoniae* to the Black Finished Sheep substrates for short time intervals. A similar phenomenon, on a smaller scale though, is observed for Beige Finished cow for all microbes. However, this antimicrobial protection extends neither to the samples incubated for 48 h nor to dual solvent dispersions. Furthermore, in general, the protection of composite TiO_2_ powders did not prove adequate in both short and long‐term experiments. Considering these data there is a profound need for higher amounts of antimicrobial factors.

## Conclusions

The results reported therein establish that the composite titania powders bearing hybrid xerogels have the potential to formulate functional coatings for medical leathers in the dual solvent dispersions used in the typical industrial process. Moreover, their production is accomplished in water at ambient temperature. Therefore, they are cheap and environmentally harmless.

Except for the incompatibility with BAC, which most probably induces pore clogging, the employment of SETAFIX as an additional solvent tends to enhance the water permeability of the more lipophilic sheep leathers while the polarity of more hydrophilic cow analogs is not compromised. The homogeneity and thickness of the composite TiO_2_ layers are also enhanced. It has even been discovered that it produces some unexpected beneficial effects by protecting the underlying lamellae of the leather from degradation and decolorization when it is immersed in water.

When exposed to low (4×10^2^ cfu/ml) to medium (4×10^3^ cfu/ml–4×10^4^ cfu/ml) concentrations of pathogen colonies dual solvent dispersions provide equal or even better protection, especially for the Gram‐positive *Staphylococcus aureus*. They do not manage though to adequately prevent microorganism adhesion and proliferation when exposed to higher concentrations (5×10^5^ cfu/ml). This outcome highlights the need for higher concentrations of antimicrobial ingredients, preferably Ag NPs at the surface of the substrates. This may be accomplished in two ways: By increasing the xerogel content of TiO_2_ composites formulation and/or by increasing the overall quantity of the coatings. However, the improved formulations and deposition methods must not undermine the leather‘s hydrophilicity.

The dispersion powders described in this work are not simply alternatives to the standard finishing process. They also represent the basis for developing additional compositions that enhance other advantageous leather properties. Silica xerogels may incorporate other active ingredients instead of BAC. Early experiments with stearic acid exhibited increased water vapor permeability. The xerogels may in turn incorporated into other ceramics. Silica Ag‐NP composites with zinc oxide increase abrasion resistance. These novel materials will be the subject of further research.

## Experimental Section

### Materials

Benzalkonium chloride in a 50 % solution form, branded as ACTICIDE® BAC 50, was acquired from the Thor Company (Wincham Northwich, UK). Titanium dioxide, powder type TiO_2_ RC 823, was purchased from CINKARNA CELJE (Celje Slovenia). Ampicillin, C_16_H_19_ N_3_O_4_S, Tetraethyl orthosilicate, Si(OC_2_H_5_)_4_, and silver nitrate, AgNO_3_, were procured from Sigma‐Aldrich Steinheim, Germany. Trizma base, NH_2_ C(CH_2_OH)_3_, was bought from Research Organics (Cleveland, OH, USA). PEI‐25,000 (MW 25,000), traded under the name Lupasol WF, was sourced from BASF (Ludwigshafen, Germany).Additionally, 1‐methoxy‐2 propanol, CH_3_CH(OH)CH_2_OCH_3_ (PM), was sourced from SHELL Chemicals (Shell Chemicals Europe BV, Rotterdam /Pernis, Netherlands) and nitrocellulose solution, trade name SETAFIX, from Fenice S.pA (Valdagno Vicenza Italy). The aqueous solutions were prepared with ultrapure water having a conductivity of 18.2 M cm, which was sourced employing the Millipore Milli‐Q system (Millipore, Bedford, MA, USA). All reagents were used as received.

### Instrumentation

The hydrophilicity of the leather coupons was assessed by measuring the dynamic water contact angle (CA) on a Kruss DSA30S instrument (Hamburg, Germany), having a surface tension range of 180 degrees, spanning from 0.01 to 2000 mN/m. The digital images were recorded by the Advance‐Kruss 1.5.1.0 software which also performed the calculation of contact angles. The depiction of the samples in the microscale was realized by low‐vacuum scanning electron microscopy (SEM) on the FEI Quanta Inspect microscope equipped bearing a tungsten filament, (FEIH Hillsboro, OR, USA) and a Thermo Fisher Scientific Talos F200i S/TEM Transition Electron Microscope equipped by EDX Bruker 6|T Flash 100 mm^2^. An estimation of the chemical composition of the final processed products derived from the additional energy‐dispersive X‐ray spectroscopy (EDS) experiments. To track the diffusion of chemical substances from both untreated and treated leather samples, a Cary 100 UV‐visible spectrophotometer I from Varian Inc. in Palo Alto, CA, USA, was employed.

### Sheep and Bovine Hides Anterior Processing

The raw sheep hides were obtained from animals living in the Peloponnesus district the southern part of mainland Greece and had an average surface area of approximately 1 m^2^. The cow analogs, with an average surface area measuring 3.7 m^2^, came from species from Macedonia the northernmost region of the same country. The preservation of all specimens was made with common NaCl and then a standard wet blue leather production method was followed with minor adjustments in the methodology focused mainly on the alteration of some reagents that are the most suitable for each type of leather. Chromium (III) sulfate ([Cr(H_2_O)_6_]_2_(SO_4_)_3_) was used for the initial tanning whereas the re‐tanning stage was, carried out using sodium aluminosilicate AlNa_12_SiO_5_ and/or MgO. All stages of the process i. e. a) soaking, b) liming, c) deliming d) bating, e) pickling, f) tanning, g) re‐ tanning, h) fat‐liquoring, and i) dyeing were executed in typical laboratory drums.

### Formation of the Silicon Oxide‐PEI‐Ag Nps Xerogel Powder (PEI‐Ag‐SiO_2_)

A solution of hyperbranched PEI with a total concentration of 40 mM, in primary and secondary amino groups (0.12 gr of the polyamine in 50 ml deionized water). After approximately one hour, the introduction of 12.5 mL of 0.1 M AgNO_3_ created a light‐yellow hue, signifying the onset of AgNps nucleation. Their development was completed after 8 days, along with a chromatic transition to reddish‐brown.[^35^, ^36^] An equivalent volume of 1 M orthosilic acid was then added. The latter was synthesized by acid hydrolysis of 10.4 gr tetraethoxysilane with 250 μL HNO_3_. The solidification of the gel precursor solution was accelerated by pH adjustment to 7.5 employing trizma base and took place within two hours. Conversion to xerogel was performed by 5 days of drying at 60 °C and then a second dehydration step in the presence of P_2_O_5_, under vacuum.

### Formation of the Titanium Oxide‐Silicon Oxide‐PEI‐Ag Nps Xerogel Powder (PEI‐Ag‐SiO_2_‐TiO_2_)

The procedure for the hydrogel is identical to that followed in the previous paragraph. Immediately after the pH fixation, i. e. still in the liquid state, 20 mL were admixed with 30 g of TiO_2_ powder. The liquid gel precursor solution is absorbed by the ceramic powder indicating that a substantial part is entering the pores of the ceramic substrate. The homogenization into the pores of the ceramic was facilitated by gentle steering with a spatula, ultimately creating a paste. By this method, coagulation also occurs in the interior of the titania particles, and the gel is integrated effectively. The subsequent drying steps were in line with the previously described two‐step technique.

### Formation of the Titanium Oxide‐Silicon Oxide‐PEI‐BAC Xerogel Powder (PEI‐BAC‐SiO_2_‐TiO_2_)

In this case, 50 ml of 1 M orthosilicic acid was merged with the same quantity of the above‐mentioned PEI solution and was combined at pH 7.5 with 30 g of TiO_2_ as reported hereinabove. After undergoing a similar gelation path and subsequent drying, the addition of 20 mL of 50 % BAC 50 solution followed, transforming the white powder into a paste resubmitted to an identical drying process.

### Formation of the Titanium Oxide‐Silicon Oxide‐PEI‐Ag Nps‐BAC Xerogel Powder PEI‐Ag‐BAC‐SiO_2_‐TiO_2_

The protocol implemented for the assembly of the xerogel powder bearing both antimicrobial agents (silver NPs and BAC) closely resembles the one disclosed in the previous paragraph. Notably the aqueous PEI 25,000 solution, is replaced with the PEI containing Ag Nps that was prepared as detailed in the relevant synthetic pathway.

### Coating of the Leathers by the Xerogels

The treatment of the substrates proceeded according to a usual finishing process, typically used in the industry. More specifically the dispersions were formed by adding 1.5 g of the xerogels into 45 g of PM. 50 g of the SETAFIX microemulsion was also diluted in 50 mL of deionized water. The two liquids were brought together and the mixture was introduced into a spray gun equipped with an air compressor (Springfield Leather Company, Springfield, MO, USA) and applied to the leather samples at a rate of 20 mL/cm^2^ of leather. For comparison purposes, the procedure was also repeated using only the initial dispersions of the xerogel powders in PM. Since the latter were about twofold denser the volume of the dispersion sprayed was halved. The two different leather varieties and the different practices are summarized in Table 1

**Table 1:** Categorization of the two leather types and the two different processing methods with the four different xerogel dispersions.

Leather Type/Treatment	Black Finished Sheep	Beige Finished Cow	Beige Finished Cow PM/	Black Finished Sheep PM/
	PM	PM	SETAFIX	SETAFIX
Blind (unprocessed)	1	2	2	1
PEI‐BAC‐SiO_2_‐TiO_2_	A1	A2	A3	A4
PEI‐Ag‐SiO_2_‐TiO_2_	B1	B2	B3	B4
PEI‐Ag‐BAC‐SiO_2_‐TiO_2_	C1	C2	C3	C4
PEI‐Ag‐SiO_2_	D1	D2	D3	D4

### Assessment of Antibacterial Potential

The disk diffusion method was selected and applied to *Escherichia coli, Staphylococcus aureus, and Pseudomonas aeruginosa* to estimate the coatings′ antibacterial properties. Their cultivation was carried out overnight at 37 °C in Luria–Bertani (LB) medium at 200 rpm with the aid of an orbital shaker Afterwards, the bacteria were diluted in LB agar (0.8 % w/v) to three concentrations 4×10^2^, 4×10^3^ and 4×10^4^ CFUs/mL and were layered on plates containing the same nutrient medium.

Each side of every piece of leather (0.5 cm×0.5 cm) was sterilized for 15 minutes using UV irradiation at 254 nm. The test coupons were subsequently immersed in the bacteria‐containing agar. Ampicillin was assigned as positive control whereas deionized water was the negative control. The samples were submitted to a second incubation period at the same conditions and time frame. Afterward, clear halos representing regions that inhibited bacteria proliferation were observed around each specimen. The quantification of antibacterial effectivity was performed based on the diameters of these quasi‐circular areas.

### Evaluation of the Microbicide and Anti Adherence Properties

The formulation‘s effectiveness was evaluated against *Enterococcus Faecalis, Klebsiella Pneumoniae, Candida Albicans*, and *Staphylococcus aureus* in a subsequent test. Triplicate species similar to the previous experiment were deposited in 6‐well plates containing 4 mL of 5×10^5^ CFU/mL, microbial inoculums. On this occasion, the cultures were incubated for 2 hours. After the decantation of the supernatants, the leather substrates were washed with 1 % PBS, and sonicated to extort and separate the microbe colonies that were eventually counted to assess the microbes′ affinity to the test substrates.

### Evaluation of the Antibiofilm Potential

The evaluation of activity against biofilm formation proceeded according to the same protocol as the anti‐adherence testing with the sole differentiation of a prolonged 2‐day incubation.

## Conflict of Interests

The authors declare no conflict of interest.

1

## Supporting information

As a service to our authors and readers, this journal provides supporting information supplied by the authors. Such materials are peer reviewed and may be re‐organized for online delivery, but are not copy‐edited or typeset. Technical support issues arising from supporting information (other than missing files) should be addressed to the authors.

Supporting Information

## Data Availability

The data that support the findings of this study are available from the corresponding author upon reasonable request.
