# Supplementary material for: Solvent Effect on Antimicrobial Hydrophilic Xerogel Coating of Medicinal Leathers in Simulated Industrial Finishing Process
Source: Chempluschem. 2025 Feb 21;90(5):e202400648. doi: 10.1002/cplu.202400648 (PMC12105456; doi:10.1002/cplu.202400648)
Supplement: Supplementary file 1 — Supporting Information [file CPLU-90-e202400648-s001.pdf]

ChemPlusChem

Supporting Information

Solvent Effect on Antimicrobial Hydrophilic Xerogel Coating of Medicinal Leathers in Simulated Industrial Finishing Process

Theofanis Bompotis, Eirini Karastergiou, Konstantinos Giannakopoulos, Evangelos P. Favvas, Marina Arvanitopoulou, Konstantinos Arvanitopoulos, Labros Arvanitopoulos, Georgia Kytherioti, Michail Vardavoulias, Dimitrios A. Giannakoudakis, Laura Castellsagués, Sara Maria Soto González., and Michael Arkas*

Solvent Effect on Antimicrobial Hydrophilic Xerogel Coating of Medicinal Leathers in Simulated Industrial Finishing Process

Theofanis Bompotis,[a] Eirini Karastergiou,[a] Konstantinos Giannakopoulos,[a] Evangelos P. Favvas,[a] Marina Arvanitopoulou,[a] Konstantinos Arvanitopoulos,[b] Labros Arvanitopoulos,[b] Georgia Kytherioti,[c,d] Michail Vardavoulias,[e] Dimitrios A. Giannakoudakis,[f] Laura Castellsagués,[g] Sara Maria Soto González[g,h] Michael Arkas*[a]

Supporting information

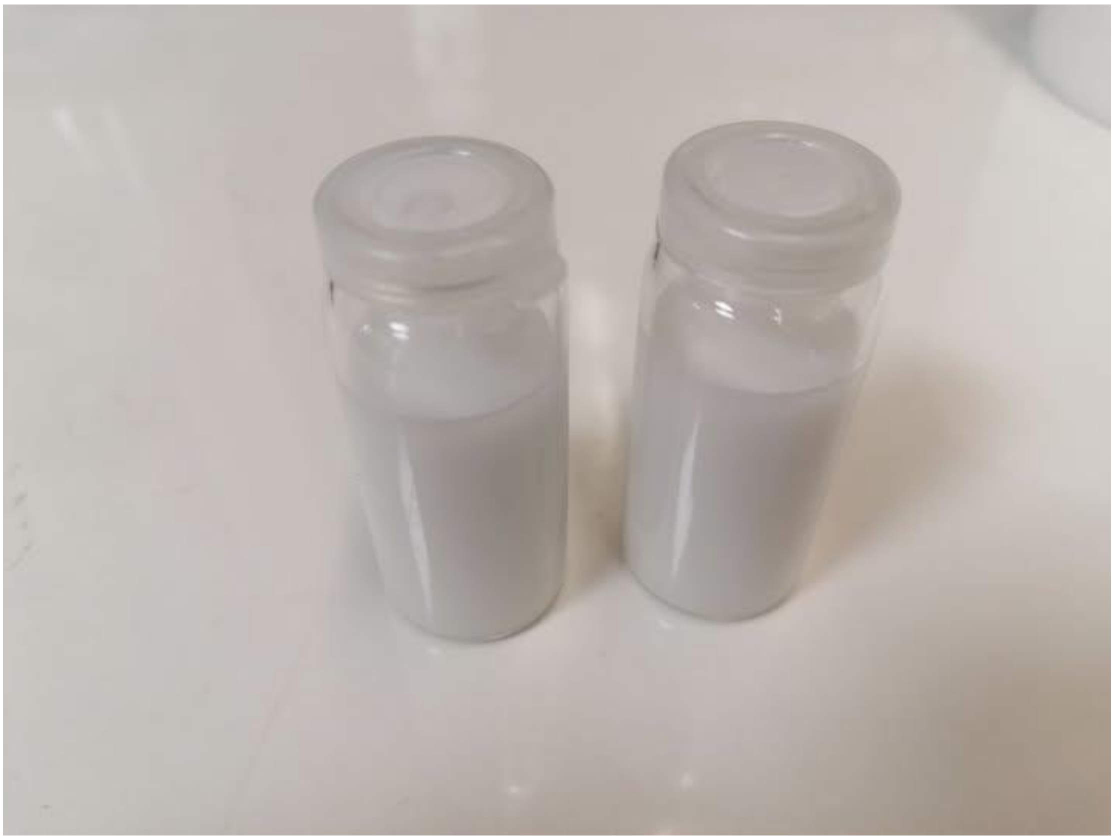

Figure S1. Comparison of non-irradiated PEI-Ag-SiO_2-TiO_2 dispersion (left) with a dispersion after irradiation at 252 nm for 1 hour (right).

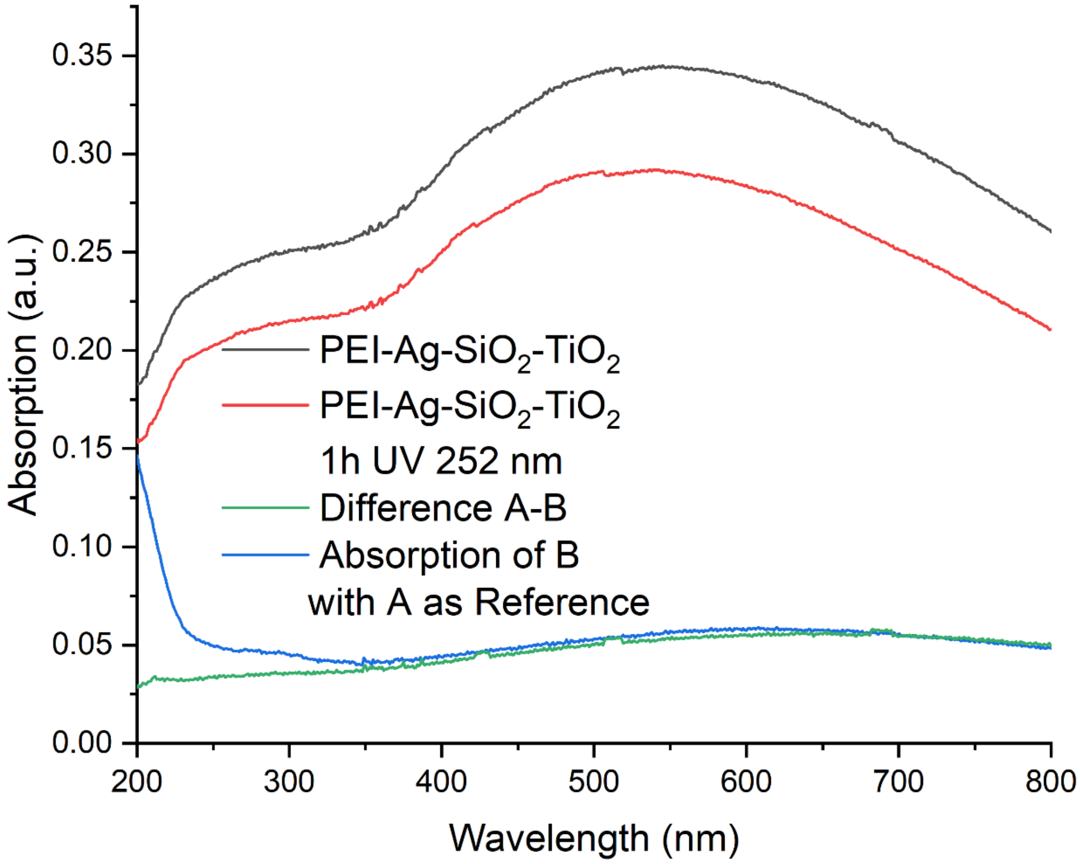

Figure S2. UV spectra of non-irradiated PEI-Ag-SiO_2-TiO_2 dispersion (black line); PEI-Ag-SiO_2-TiO_2 dispersion irradiated at 252 nm for 1 hour (red line); Subtraction of irradiated spectrum from the non-irradiated (green line); the spectrum of the non-irradiated specimen with the irradiated specimen as the reference sample.
